# Supplementary material for: mTOR and STAT3 Pathway Hyper-Activation is Associated with Elevated Interleukin-6 Levels in Patients with Shwachman-Diamond Syndrome: Further Evidence of Lymphoid Lineage Impairment
Source: Cancers (Basel). 2020 Mar 5;12(3):597. doi: 10.3390/cancers12030597 (PMC7139896; doi:10.3390/cancers12030597)
Supplement: Supplementary file 1 [file cancers-12-00597-s001.pdf]

Supplementary Materials: mTOR and STAT3 Pathway Hyper-activation is Associated with Elevated Interleukin-6 Levels in Patients with Shwachman-Diamond Syndrome: Further Evidence of Lymphoid Lineage Impairment

Antonio Vella, Elisabetta D'Aversa, Martina Api, Giulia Breveglieri, Marisole Allegri, Alice Giacomazzi, Elena Marinelli Busilacchi, Benedetta Fabrizzi, Tiziana Cestari, Claudio Sorio, Gloria Bedini, Giovanna D'Amico, Vincenzo Bronte, Antonella Poloni, Antonio Benedetti, Chiara Bovo, Seth J. Corey, Monica Borgatti, Marco Cipolli and Valentino Bezzerri

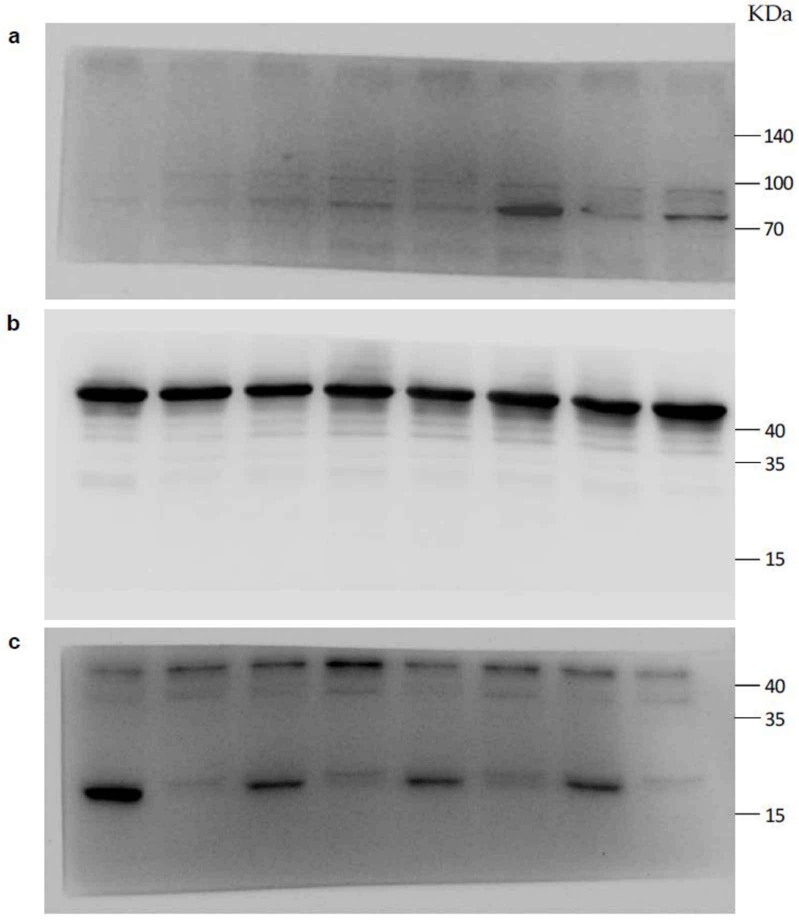

Figure S1. Whole blots showing all the bands with all molecular weight markers on the Western blot reported in Figure 3b. (**a**) Upper portion of nitrocellulose membrane (STAT3 88KDa). (**b**,**c**) Lower portion of nitrocellulose membrane (Actin 42 KDa and SBDS 28.6 KDa, respectively). The membrane was cut at ~50 KDa before primary antibody staining in order to minimize the antibody loads.

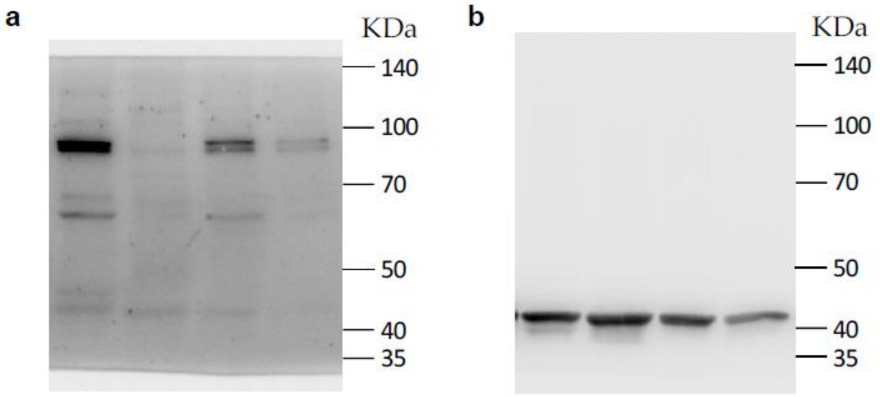

Figure S2. Whole blots showing all the bands with all molecular weight markers on the Western blot reported in Figure 4b. (**a**) STAT3 88 KDa. (**b**) Actin 42 KDa.

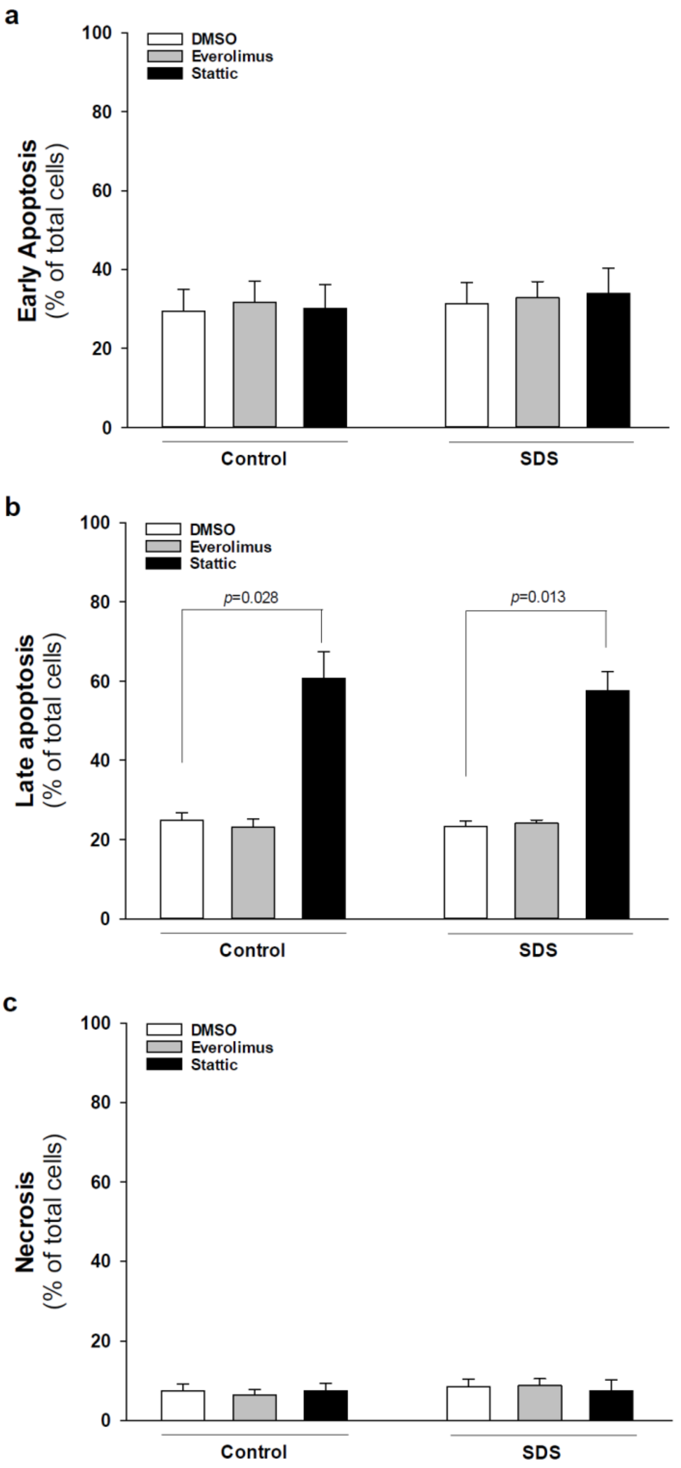

Figure 3. Apoptosis assay in LCL. LCL derived from patients with SDS or healthy controls were treated with 350 nM everolimus (grey bars) or 7.5 µM stattic (black bars) for 24 h. Apoptotic rate was detected by Dead Cell Apoptosis kit with Annexin V FITC and PI for flow cytometry. (**a**) Early apoptotic cells (Annexin-V positive, PI negative events). (**b**) Late apoptotic cells (Annexin V-positive, PI positive events). (**c**) Necrotic cells (Annexin V-negative, PI positive events). Data are mean ± SEM of 3 experiments performed in duplicate. Statistics: normal distribution was tested by the Shapiro-Wilk test. Subsequently, Mann-Whitney Rank Sum Test was calculated and reported within histograms.

Supplementary Methods

Apoptosis assay

Dead Cell Apoptosis kit with Annexin V FITC and PI for flow cytometry (Invitrogen, Carlsbad, CA, USA) was used following manufacturer's protocol. Briefly, LCL derived from SDS patients and healthy donors were incubated in the presence or absence (DMSO) of 350 nM everolimus or 7.5 μM 、 stattic for 24 h in RPMI-1640 medium supplemented with 10% FBS (Biosera, Kansas City, MO, USA). 5 μL of FITC-conjugated Annexin V were added to 100 μL of the cell suspension and incubated for 15 min at RT. Cells were then resuspended in 200 μL and 5 μL of propidium iodide were added to cells. Fluorescence signals were acquired using FACS Canto II (BD Biosciences, Franklin Lakes, NJ, USA) analyzer. A tube of unstained cells was run as internal control. Positive events for either Annexin V only (early apoptosis) or Annexin V and PI double-positive (late apoptosis) or positive for PI only (necrosis) were considered.
